# Bovine Tuberculosis: Its Communicability

**Published:** 1887-06

**Authors:** 


Bovine Tuberculosis: Its Communicability.—Dr. M. D. Blaine, (*Medical Record*, January 15, 1887,) from a study of tuberculosis in the bovine species, concludes:

1. In the bovine species, the disease is inherited either from male or female.

2. Tuberculosis is acquired by the inhalation of tuberculous substances.

3. Tuberculosis is acquired by the injestion of milk of tuberculous cows, when the disease has reached the stage of suppuration, or when there is a tuberculous affection of the milk-bag.

4. The disease may also be acquired by the injestion of the flesh of tuberculous animals.

